# Silk: Optical Properties over 12.6 Octaves THz-IR-Visible-UV Range

**DOI:** 10.3390/ma10040356

**Published:** 2017-03-28

**Authors:** Armandas Balčytis, Meguya Ryu, Xuewen Wang, Fabio Novelli, Gediminas Seniutinas, Shan Du, Xungai Wang, Jingliang Li, Jeffrey Davis, Dominique Appadoo, Junko Morikawa, Saulius Juodkazis

**Affiliations:** 1 School of Science, Faculty of Engineering and Industrial Sciences, Swinburne University of Technology, Hawthorn, VIC 3122, Australia; xuewenwang@swin.edu.au (X.W.); fabio.novelli@ruhr-uni-bochum.de (F.N.); gdseniutinas@gmail.com (G.S.); jdavis@swin.edu.au (J.D.); saulius.juodkazis@gmail.com (S.J.); 2 Department of Laser Technologies, Center for Physical Sciences and Technology, Savanoriu Ave. 231, LT-02300 Vilnius, Lithuania; 3 Tokyo Institute of Technology, Meguro-ku, Tokyo 152-8550, Japan; ryu.m.ab@m.titech.ac.jp; 4 Australian Future Fibres Research and Innovation Centre, Institute for Frontier Materials, Deakin University, Geelong, VIC 3220, Australia; shand@deakin.edu.au (S.D.); xungai.wang@deakin.edu.au (X.W.); jingliang.li@deakin.edu.au (J.L.); 5 Australian Synchrotron, Blackburn Road, Clayton, VIC 3168, Australia; dom.appadoo@synchrotron.org.au; 6 Melbourne Centre for Nanofabrication, the Victorian Node of the Australian National Fabrication Facility, 151 Wellington Rd., Clayton, VIC 3168, Australia

**Keywords:** silk, fibroin, biopolymer, terahertz, spectroscopy, solubility, proteins, biodegradable polymers

## Abstract

Domestic (*Bombyx mori*) and wild (*Antheraea pernyi*) silk fibers were characterised over a wide spectral range from THz 8 cm${}^{-1}$ (λ= 1.25 mm, f= 0.24 THz) to deep-UV $50\times 10^{3}$ cm${}^{-1}$ (λ= 200 nm, f= 1500 THz) wavelengths or over a 12.6 octave frequency range. Spectral features at β-sheet, α-coil and amorphous fibroin were analysed at different spectral ranges. Single fiber cross sections at mid-IR were used to determine spatial distribution of different silk constituents and revealed an α-coil rich core and more broadly spread β-sheets in natural silk fibers obtained from wild *Antheraea pernyi* moths. Low energy T-ray bands at 243 and 229 cm${}^{-1}$ were observed in crystalline fibers of domestic and wild silk fibers, respectively, and showed no spectral shift down to 78 K temperature. A distinct 20±4 cm${}^{-1}$ band was observed in the crystalline *Antheraea pernyi* silk fibers. Systematic analysis and assignment of the observed spectral bands is presented. Water solubility and biodegradability of silk, required for bio-medical and sensor applications, are directly inferred from specific spectral bands.

## 1. Introduction

Spectral properties at sub-1 mm wavelengths at around and below terahertz frequencies (1 THz = $10^{12}$ Hz, corresponding to ≈33 cm${}^{-1}$ in wavenumbers) are important for understanding materials with bio-medical relevance [1]. For example, terahertz absorbance is related to conformation and structure of saturated fatty acids with long alkane chains, whose vibration frequency depends on the length *l* of the chain as $\nu =\sqrt{\frac{E}{\rho }}/\left(2lc\right)$ [cm${}^{-1}$] for density ρ, Young modulus *E*, and speed of light *c*—as demonstrated for polymethylene [2]. Those longitudinal accordion modes (LAMs) populate a 1–100 cm${}^{-1}$ low energy vibration window, edging towards low wavenumbers for long chains. Of relevance to material science are first order solid–solid phase transitions in alkane crystals—the rotator phases [3]—occurring just a few degrees below melting point when the crystalline order still exists; however, low energy rotations of molecules become allowed [4]. In glasses, low frequency Raman spectra exhibit the 10–50 cm${}^{-1}$ Boson peak, which is another example of low energy phenomena due to rearrangement of density of states in amorphous materials [5].

In protein based materials, a variety of molecular ordering and interactions occur, which, in turn, define their properties. It was shown that formation of protein fibrils can be monitored at the THz spectral window [6]. Silk fibers offer a good example of the complexity of protein materials as they have amorphous and crystalline structural components with proteins forming a 3D network of random α-coils and metastable β-turns (Silk I) together with a crystalline β-sheet phase (Silk II) [7]. Such composition results in a set of important properties such as a high mechanical strength, optical transparency and waveguiding [8] as well as biocompatibility and biodegradability [9,10].

Mechanisms controlling crystallisation of protein coils via ordered hydrogen bonding and their unzip-decomposition at different annealing temperatures, laser and electron beam exposure conditions are of interest for applications in material science and bio-medical fields [11,12,13]. Silk can be used as a bio-compatible scaffold [14] with water solubility dependent on its crystallinity [15]. Furthermore, the biodegradability and elasticity of silk make it an attractive platform for the creation of next-generation biocompatible and flexible optoelectronic devices [16]. Any active functional materials used in such applications alongside silk would have to exhibit conformable mechanical characteristics. Two-dimensional transition metal oxides, with their wide variety of controllable physical properties, are especially promising in this regard [17]. While the field is still nascent, silk fibroin was already demonstrated to be a viable template in preparing metal oxide composite nanomaterials for lithium-ion battery anodes [18,19]. However, in order to tune the morphology of nanomaterials created in this way, control over the self-assembly behavior of hierarchical fibroin structure is required [20].

Photo-thermal control of β-sheet formation in silk could provide a way to make silk-resists as well as 3D bio-scaffolds, and would help to understand protein crystallisation mechanisms relevant to the β-sheet plaque formation in Alzheimer’s disease. It was demonstrated using on-chip calorimetry that, through a fast 2000 K/s thermal quenching of molten silk, an amorphous phase (water soluble) can be recovered [21]. However, fast thermal quenching is hampered by the rather low thermal diffusivity of silk $\alpha_{T}≃1.5\times 10^{-7}$ m${}^{2}$/s [22], likewise attributable to its complex structure. Similarly, the interaction of light with the multiple constituent structural components of silk fibers at frequencies spanning from far-IR to UV spectral ranges has to be well understood for a wide range of applications.

Here, transmittance measurements of silk over a broad spectral range from THz wavelengths (T-ray) until deep UV are reported and spectral signatures of the constituent components of silk: β-sheets, α-coils and amorphous fibroin are analysed. Since the spectral signatures characteristic of the silk building blocks are present at very different wavelengths, a comprehensive analysis over the broad spectral range had to be made. Water solubility of silk can be inferred from the spectral properties and is essential for future applications of silk in wearable electronics, implants, and sensors.

## 2. Materials and Methods

### 2.1. Spectroscopy Setups and Techniques

The THz/Far-IR Beamline at the Australian synchrotron was used to characterise silk in the 40–600 cm${}^{-1}$ spectral range. The beamline is equipped with a Bruker IFS 125/HR Fourier Transform (FT) spectrometer (Bremen, Germany) and OPUS 6.5 software (Bruker Optik GmbH, Ettlingen, Germany) was used for initial data analysis. Up to 100 spectral scans were captured and averaged to improve signal-to-noise (S/N) ratio. A liquid nitrogen cryostat was used to measure silk transmittance, *T*, down to ∼77 K temperature.

For the largest T-ray wavelengths in the 8–80 cm${}^{-1}$ spectral window, femtosecond time domain spectroscopy (TDS) was used due to the higher S/N ratio as compared to synchrotron T-ray radiation. Almost a single-cycle and 1 ps long THz-fields are generated in a 0.6% MgO doped LiNbO${}_{3}$ crystal by optical rectification of amplified laser pulses [23,24]. The radiation is focused onto the sample by an off-axis parabolic mirror with a focal length of 100 mm and imaged with two additional identical mirrors onto the detection crystal. The single-cycle fields are detected via electro-optical sampling in a 500 μm thick ZnTe [25].

**Figure 1:**
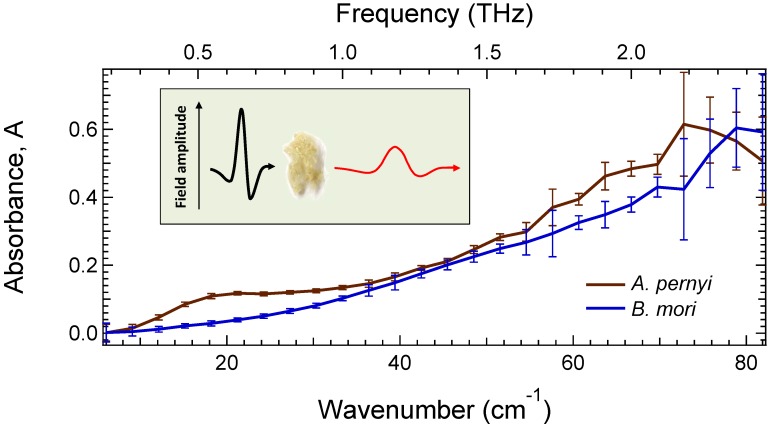
Absorbance, A=−lgT, spectra of *B. mori* and *A. pernyi* silk fibers measured by time domain spectroscopy (TDS). The transmittance, *T*, is calculated as the ratio of the magnitudes of the Fourier transforms of the field transmitted through the sample and through a reference (air). Error bars represent one standard deviation from 50 measurements.

The amplitude and phase-resolved fields transmitted through the silk samples, as well as through a reference (air), are Fourier transformed to give frequency-dependent amplitudes as well as their phases. If the sample thickness is well defined and only the first Fresnel coefficient can be considered, the phase difference of these Fourier transforms is directly related to the real part of the index of refraction, whereas the ratio of the field magnitudes yields the absorption coefficient. In the case of silk fibers, however, the sample thickness is not well defined, so only the absorbance, *A*, can be unambiguously deduced, calculated as -lgT, where *T* is the field transmittance corresponding to the ratio of the Fourier magnitudes. The fields transmitted by a reference and by the sample are alternatively acquired 50 times to give the standard deviation shown as error bars in Figure 1.

Shorter wavelength characterisation was carried out with a UV-Vis spectrometer (Lambda 1050 UV/Vis, PerkinElmer, Waltham, MA, USA) by measuring total transmittance and reflectance of fibers in a 150 mm integrating sphere geometry. Photoluminescence excitation spectra were collected using a fluorescence spectrometer LS55 (PerkinElmer), whereas a FT-IR spectrometer (Vertex70, Bruker) was used for far-IR-to-near-IR transmittance measurements. Spectral ranges of the selected tools allowed for continuously covering an unprecedentedly wide spectral range from T-rays to deep-UV.

The measured spectral properties over a large range of wavelengths have different contributions of Rayleigh scattering, which is proportional to $\lambda^{-4}$ and Mie scattering, which becomes significant for size parameter x=(2π/λ)d>1, given here for spherical particles of radius *d* for which analytical solutions are known. Mie scattering has very strong polarization and angular dependence [26], which makes absorbance measurements sensitive to the numerical aperture. Furthermore, Mie scattering has stronger intensity fluctuations for more absorbing materials. For direct estimation of Mie scattering of realistic samples, a large number of silk fibers would be required; however, this was out of the scope of the current study.

### 2.2. Silk Samples

Silk samples were prepared from domestic *Bombyx mori* and wild *Antheraea pernyi* species of silk worms. *A. pernyi* silkworm cocoons were obtained from Liaoning province, China. *B. mori* was collected from the silk rearing house in Jiangsu province, China. The silk fiber from *B. mori* has superior elasticity and toughness due to the way the disordered fibroin matrix is reinforced by glycine and alanine based β-sheets [27]. However, *A. pernyi* has a different primary structure of fibroin, composed of alternating appearances of large repetitive poly-alanine blocks and glycine rich regions [28]. This is in contrast to *B. mori* but has distinct similarities to spider dragline silk [29]. Furthermore, *A. pernyi* survives at the lowest temperatures among all silk moths. Raw cocoons were degummed three times using 0.5% Na${}_{2}$CO${}_{3}$ solution at 98 ${}^{\circ }$C for 1 h for *A. pernyi* cocoon, but 30 min for *B. mori* cocoon [30,31]. Finally, the degummed silk fibers were thoroughly rinsed with warm deionised water (60 ${}^{\circ }$C) prior to being dried in air.

Fibroin extraction from degummed *B. mori* silk fibers was done by first dissolving them in a 1:8:2 molar ratio ternary mixture of CaCl${}_{2}$/H${}_{2}$O/CH${}_{3}$CH${}_{2}$OH 65 ${}^{\circ }$C. Then, the solution was dialysed against ultra-pure water with dialysis tubing cellulose membrane (molecular weight cut-off 14 kDa, Sigma-Aldrich Co., St. Louis, MO, USA) at room temperature for 4 days. Finally, silk fibroin was regenerated by lyophilizing the dialysed solution. The same procedure was used in prior work to make a fibroin-based electron beam resist [22].

Silk fibroin samples spectroscopically investigated in this work allow for comparisons between β-sheet rich, high-crystallinity, hence water-insoluble silk fibers, and soluble amorphous fibroin without significant β-sheet content. Furthermore, fibers originating from different silkworm species exhibiting variations in secondary structure are also probed in a broad spectral range.

## 3. Results and Discussion

Different spectral ranges of silk absorbance/transmittance have been probed using different methods; however, there was always an overlap between two adjacent spectral ranges. Absorbance at the longest T-ray wavelengths at 8–80 cm${}^{-1}$ (0.24–2.4 THz; 1.25 mm–125 μm) was measured with TDS while, for the shorter wavelengths up to 600 cm${}^{-1}$ (18 THz; 16.6 μm), synchrotron beamline was used. At wavenumbers above 600 cm${}^{-1}$, investigation was conducted by means of FT-IR spectroscopy up to 4000 cm${}^{-1}$ (120 THz; 2.5 μm), at which point near-IR-to-visible spectroscopy took over up to wavenumbers of 33,333 cm${}^{-1}$ (roughly corresponding to 1000 THz in frequency and 300 nm in wavelength). Finally, photoluminescence excitation spectroscopy aided in providing information at deeper UV wavelengths up to 50,000 cm${}^{-1}$ (1500 THz; 200 nm).

### 3.1. T-Rays

The terahertz frequency range is associated with low frequency macromolecular motions strongly related to the dynamics and conformational changes of proteins and peptides [32]. Hence, it is useful in probing the secondary and to some extent the primary structure of complex biomaterials. Figure 1 shows absorbance spectra measured with the TDS technique. Distinct 20 cm${}^{-1}$ and 67 cm${}^{-1}$ (0.6 THz and 2 THz) bands were observed in *A. pernyi* silk fibers.

**Figure 2:**
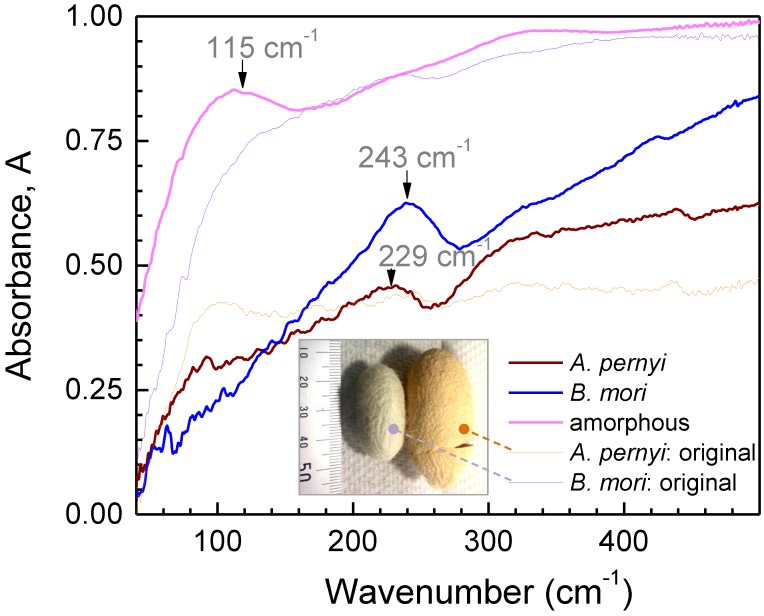
T-ray absorbance, *A*, spectra of domestic (degummed *B. mori*) and wild (degummed *A. pernyi*) silk fibers together with amorphous fibroin extracted from *B. mori* silk and fibers from non degummed, as harvested, silk cocoons with sercin. All spectra were measured at room temperature (RT). Data averaged over 100 scans.

Figure 2 shows room temperature (RT) far-IR synchrotron radiation absorbance, *A*, spectra of different silk samples. Sericin-free crystalline fibers of *B. mori* and *A. pernyi* silk have slightly different spectral positions of the absorption bands. Amorphous water soluble fibroin had a spectrally broader absorption at ∼115 cm${}^{-1}$. All the spectra were measured with fibers or film (amorphous) suspended over a hole though which a 3-mm-diameter T-ray beam was propagating. There were no artifacts due to normalization to the background T-ray transmittance at those spectral locations.

**Figure 3:**
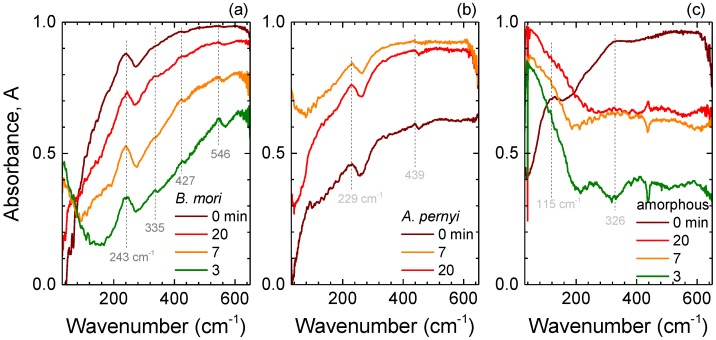
Absorbance, *A*, spectra of (**a**) *B. mori* silk fibers; (**b**) *A. pernyi* silk fibers and (**c**) amorphous silk fibroin samples annealed on a hot plate for 3, 7, 20 min at cleanroom class 1000 conditions. Amount of silk in each sample was the same (0.6 mg) during annealing; however, for IR spectral measurements, a small amount of fibers was placed over the 3-mm-diameter aperture. Data averaged over 100 scans. Temperature of the hot plate was 250 ${}^{\circ }$C.

Absorbance spectra of degummed *B. mori* and *A. pernyi* silk fibers as well as amorphous fibroin from *B. mori* subjected to hot plate annealing at 250 ${}^{\circ }$C, i.e., at the onset of thermal degradation [33], for varying durations are shown in Figure 3. Degradation of silk with observable darker coloration was consistent with high temperature oxidation. However, for the crystalline β-sheet rich fibers, there were no strong changes in the absorption bands at ∼243 ± 15 cm${}^{-1}$ (*B. mori*) and ∼229 ± 15 cm${}^{-1}$ (*A. pernyi*) silk (Figure 3a,b). This is consistent with β-sheets exhibiting a higher resilience to thermal decomposition than random coil structures. Furthermore, at elevated temperatures, metastable fibroin fractions in the secondary structure of silk are liable to transform into the stable β-sheet crystals [33]. Hence, the observed variations in T-ray absorbance spectra for the different durations of heating at the lower edge of the thermal degradation range can tentatively be related to a decrease of water content or preferential decomposition of the amorphous regions in comparison with β-sheets. Further support is provided by spectral variations due to thermal degradation in amorphous *B. mori* fibroin. It experienced a strong reduction of absorbance throughout the investigated range, especially at larger wavenumbers and a disappearance of the ∼125 ± 15 cm${}^{-1}$ band (Figure 3c).

**Figure 4:**
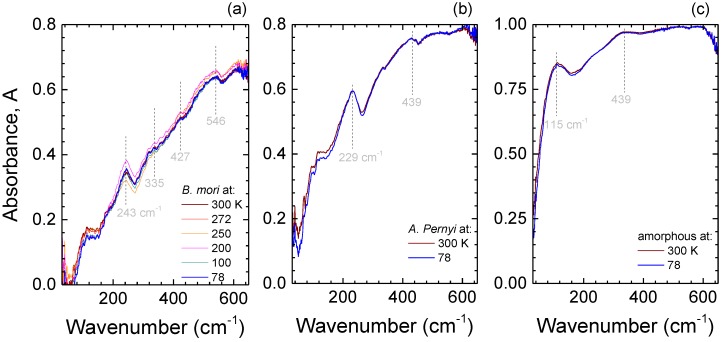
Temperature dependence of absorbance, *A*, spectra of (**a**) *B. mori* degummed silk fibers; (**b**) *A. pernyi* degummed silk fibers and amorphous (**c**) *B. mori* fibroin samples at different temperatures using a liquid nitrogen cryostat. Data averaged over 100 scans. Note different *A* scales between (**a**–**c**).

The spectral range in the vicinity of 240 cm${}^{-1}$ is also associated with water absorption bands. In particular, at room temperature, liquid water has a broad spectral feature at 200 cm${}^{-1}$, assigned to the stretching of intermolecular hydrogen bonds, and another at 700 cm${}^{-1}$, related to librational motions [34,35]. Therefore, it is important to ascertain whether the observed peaks are related to water content. As silk is heated, water removal by evaporation efficiently proceeds in the range from 70 ${}^{\circ }$C to ∼200 ${}^{\circ }$C [36]. Therefore, if the peaks at around 240 cm${}^{-1}$ were related to water, they would be expected to gradually disappear in the thermally treated samples, which was not observed in experiment (Figure 3). Conversely, water can also be revealed by ice formation and a corresponding emergence of a spectrally narrow feature at around 230 cm${}^{-1}$ when cooled towards liquid nitrogen (77 K) temperature [37,38]. The crystalline and amorphous samples were cooled and their absorbance spectra measured (Figure 4). A spectrally narrow ice band was not observed. There were no spectral shifts with lowering temperature and only slight narrowing of the characteristic absorbance bands, best recognizable in *A. pernyi* silk (Figure 4b). Thereby, water can be ruled out as the cause of the observed T-ray absorbance peaks.

The far-IR spectra of natural silk fibers are made more complex by the heterogeneous arrangement of their constituent proteins. However, for *B. mori* silk, in addition to the dominant 243 cm${}^{-1}$ absorption band, three other peaks at 335, 427, and 546 cm${}^{-1}$ can be discerned, especially for heat-treated samples with reduced water content. Four bands have been previously observed in a similar spectral vicinity at 250, 328, 427, and 553 cm${}^{-1}$, respectively, for β-sheet rich fibroin films [39]. In contrast, *A. pernyi* silk fibers have far-IR absorbance spectra with two discernible peaks—at 229 and 439 cm${}^{-1}$. The prevalence of poly-alanine in wild silks provides a hint to their possible assignments. Stretched β-form of poly-l-alanine exhibits absorbance peaks at 240 and 432 cm${}^{-1}$ [39]. Lastly, amorphous *B. mori* fibroin spectra have a broad peak at ∼115 cm${}^{-1}$ as well as a shoulder at 326 cm${}^{-1}$, consistent with the α-helix state of poly(alanine-glycine) (Ala-Gly) [39]. In addition, the observed variations of these spectral features due to heat treatment would lend further credence to their assignment to the metastable Silk I state.

Overall, the peaks observed in the fibers at far-infrared exhibit slight shifts with respect to their extracted counterparts; furthermore, they are generally broad and not well defined. This lends credence to the view that, at the terahertz range, ensembles of resonances characterising the secondary structure of proteins are detected [40]. This, in turn, opens new possibilities to probe structural properties at T-ray bands beyond the scope of typical spectroscopic approaches.

### 3.2. Mid-IR and IR Range

**Figure 5:**
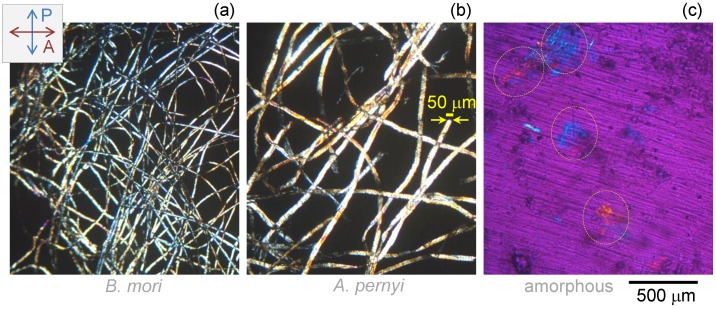
Optical cross-polarised optical images of degummed fibers of *B. mori* (**a**); *A. pernyi* (**b**); and amorphous fibroin from *B. mori* silk (**c**). Samples were compressed with KBr powder to form ∼100 μm thick pallets. Marked regions in (**c**) show locations of amorphous fibroin. An additional λ/2-plate was introduced to set the best background contrast with the same color corresponding to the same difference in the optical path length.

The spectral range in excess of 600 cm${}^{-1}$ wavenumbers, beyond synchrotron T-ray scope, was probed using mid-IR FT-IR spectroscopy and imaging. Fibroin based fibers have been extensively studied in this wavelength region [41,42]; therefore, measurements provide a way for verification against the established body of work. Figure 5 shows cross polarised images of the samples. Degummed *A. pernyi* silk fibers were ∼50 μm in diameter and approximately 2–3 times thicker than *B. mori* silk fibers.

**Figure 6:**
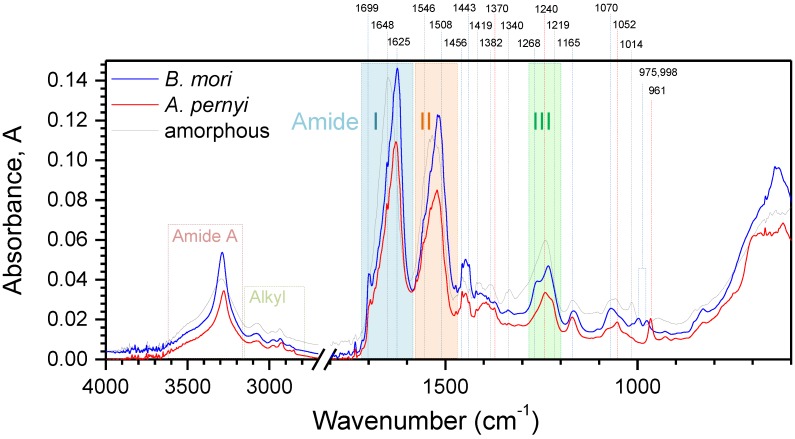
Absorbance, *A* spectra of *B. mori* and *A. pernyi* silk fibers and amorphous fibroin powder measured using a diamond window and attenuated total reflection (ATR) method (Alpha, Bruker).

Figure 6 shows mid-IR spectra of domestic *B. mori* and wild *A. pernyi* species’ silk fibers as well as of extracted fibroin, taken using the attenuated total reflection (ATR) technique. The N–H stretching, alkyl, and Amide bands represent the most prominent spectral features for all three samples. Some absorbance peaks of significant interest are the 961 cm${}^{-1}$ band attributed to poly-alanine (Ala)${}_{n}$ in β-sheets, observed primarily in wild silks [41]. Conversely, domesticated *B. mori* derived white silk fibers exhibit different, slightly subdued features at 975 and 998 cm${}^{-1}$, consistent with their (Ala-Gly)${}_{n}$ based β-sheets [41,43]. The component at 1014 cm${}^{-1}$ is associated with random coil (Ala-Gly) sequences that have many interruptions by tyrosine (Tyr), valine (Val) or other peptide chains [43], and, as expected, it is most pronounced in the spectrum of disordered amorphous fibroin. Peaks in the vicinity of 1052 cm${}^{-1}$ fall within the ν(C–C) skeletal range and are expected to have variations in different silks due to the presence of small amounts of other amino-acids in addition to Ala and Gly—with the 1052 cm${}^{-1}$ peak being more closely associated with α-coils and metastable β-turns (i.e., Silk I), whereas 1052 cm${}^{-1}$ was shown to be related to anti-parallel β-sheets (Silk II) [43]. The band at 1165 cm${}^{-1}$ is common among all silks and assigned to ν(N–C${}_{\alpha }$) vibrations [41]. Its width is generally larger for domesticated silk species and even more so for amorphous fibroin, indicating more variety in conformation states.

The Amide III band ranging from 1200 cm${}^{-1}$ to 1300 cm${}^{-1}$ is a complex spectral region with overlapping contributions from various amide side-chains in differing conformations [44]. The components are typically assigned for β-sheets at 1219 cm${}^{-1}$, random coil at 1240 cm${}^{-1}$ and α-coils at 1268 cm${}^{-1}$ [41], with *B. mori* exhibiting a peak at 1232 cm${}^{-1}$ consistent with the Silk I form. Finally, a feature at 1308 cm${}^{-1}$ is generally associated with β-turns [41]; however, in the present case, the signature is weak. The amorphous fibroin spectrum has a broader, less defined line at the Amide III band, with more spread towards higher wavenumbers, in line with its disorder. Likewise, amorphous fibroin and, to a lesser extent, *B. mori* silk has a strong line at 1340 cm${}^{-1}$ due to $\delta_{s}$(CH${}_{3}$) also associated with the Silk I form [43]. Signatures in the 1350–1420 cm${}^{-1}$ window likewise represent bending vibrations $\delta_{s}$(CH${}_{3}$) for polypeptides in various conformations [45]. The spectral line at 1443 cm${}^{-1}$ corresponds to asymmetric bending vibrations of $\delta_{as}$(CH${}_{3}$) variety in β-sheets of both poly-alanine (Ala)${}_{n}$ and Ala-Gly${}_{n}$ [41], with intensity being highest in β-sheet rich domestic white silk and lowest for amorphous fibroin. The similarly $\delta_{as}$(CH${}_{3}$) associated line at 1456 cm${}^{-1}$ is related to the more generic vibrations in alanine and valine.

The three distinct varieties of fibroin-based structures show distinct variations in the Amide I and Amide II regions that dominate the spectra. Each of these lines has a complex spectral composition due to conformational variations [43]. The major constituents of the Amide II band are assigned as 1508 cm${}^{-1}$ arising from β-sheets and 1546 cm${}^{-1}$ is associated with disordered fibroin, as is evidenced by its prevalence for the amorphous sample. Amide I band follows a similar distribution but shows an even more pronounced structure, with components at ∼1625 cm${}^{-1}$ representing β-sheets and 1648 cm${}^{-1}$ associated with irregular structures including random coil and extended chains [43]. In addition, other signatures reported in literature are associated with Silk I, type II β-turns (1647–1654 cm${}^{-1}$), α-coils (1658–1664 cm${}^{-1}$) and turns and bends 1699 cm${}^{-1}$ [46] (unaffected by the β-sheet disrupting fibroin extraction procedure). The Amide I, II bands were found broader in transmission (not shown) for the fibers as compared with amorphous fibroin [22], which is consistent with a higher orientation arising due to drawing [47]. In the single beam transmittance, the signal is integrated across the diameter of the fiber, whereas the ATR signal penetrates only a few micrometers from the surface. It is well known that the silk fiber has a core and shell structure, and the shell is comprised of low molecular weight amides while the core is rich in extended (Ala-Gly)${}_{n}$ or (Ala)${}_{n}$ chains [47]. Differences between degummed crystalline fibers of domestic (white) and wild (brown) silk are the most distinguishable around the 1000±100 cm${}^{-1}$ region. It was likewise previously observed that strong polarisation dependence exists at this wavelength range suitable for identification of silk species [31].

**Figure 7:**
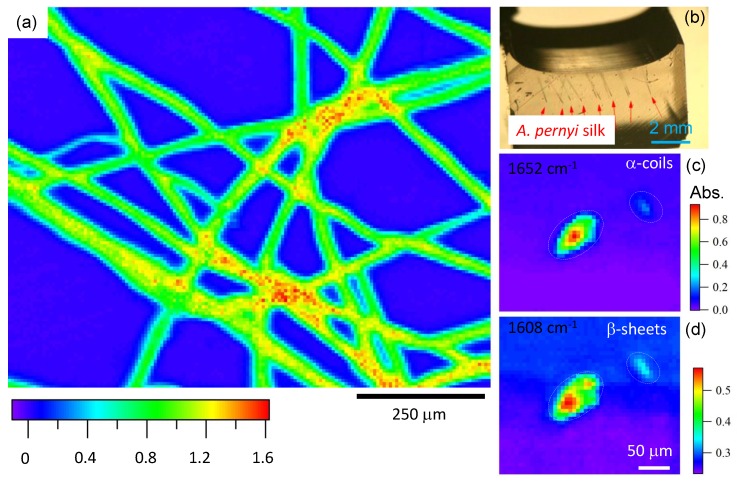
(**a**) natural *A. pernyi* silk absorbance IR image taken at ∼1660 cm${}^{-1}$ band (the region of α-coils absorption); (**b**) photo of silk fibers embedded in a block of epoxy which was used for 5-μm-wide microtome slices; (**c**,**d**) absorbance: axial cross sectional scans of *A. pernyi* fiber at β-sheet (1608 cm${}^{-1}$) and α-coils (1652 cm${}^{-1}$) bands. Resolution was 6.25 μm. Oval contours show circumference of the fibers.

In order to shed further light on the internal structure of silk transmittance, IR imaging was performed for natural (not degummed) sericin rich brown silk fibers embedded in a thin KBr pallet. Spectroscopic maps were acquired at the 1660 cm${}^{-1}$ absorbance band preferentially caused by α-coils, which were shown to be located towards the center of the fiber (Figure 7). The fiber is clearly distinguished from the KBr matrix. Furthermore, for the cross-sectional observation, the natural silk fibers were aligned and embedded into an epoxy adhesive (jER 828, Mitsubishi Chemical Co., Ltd., Kyoto, Japan) as shown in the Figure 7b. Fibers fixed in the epoxy matrix were cut in the perpendicular direction to the fiber by microtome (RV-240, Yamato Khoki Industrial Co., Ltd., Saitama, Japan). Figure 7c,d shows mid-IR transmittance spectral imaging of the ∼100 μm-diameter fiber and a smaller whisker (oval contours) taken at different wavenumbers. The difference in distribution at 1608 cm${}^{-1}$ and and 1652 cm${}^{-1}$ absorption bands of β-sheets and α-coils, respectively, is clearly distinguishable. The α coils are located preferentially at the center of the fiber (as inferred from Figure 7), while β-sheets are distributed over the entire cross section and are present at the rim. This is consistent with observed differences between transmission and ATR measurements of silk absorbance showing slightly different absorption losses due to, respectively, throughout and evanescent propagation of light in those two modes of measurement [47].

### 3.3. Vis-UV Range

**Figure 8:**
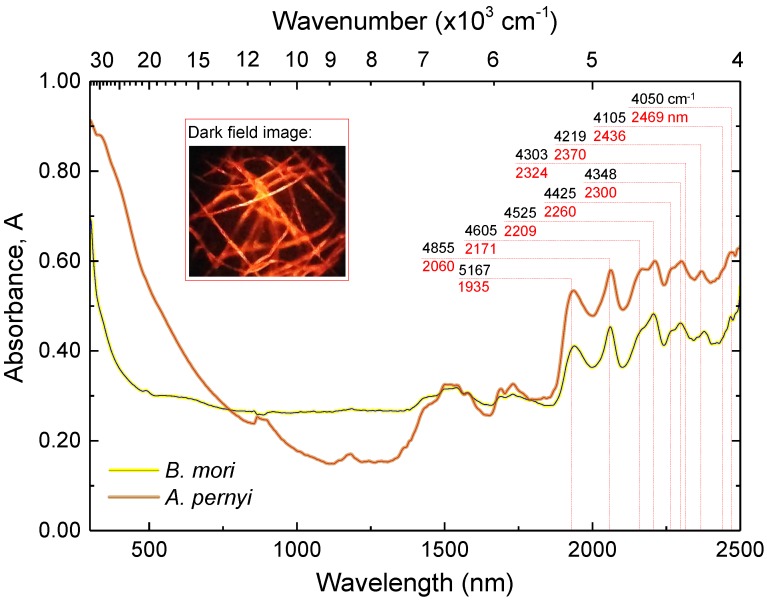
UV-near-IR Absorbance, *A*, spectra of degummed fibers of *B. mori* and *A. pernyi*. Inset shows an optical dark-field image of silk fibers. See text for the band assignment.

Fibroin based compounds have a low molar absorptivity in the visible to near-infrared spectral regions. Furthermore, the effects of water vapour are negligible at such wavelengths. Figure 8 shows the absorbance of domestic *B. mori* and wild *A. pernyi* silk fibers from the shortest UV wavelengths to near-IR spectral range, deduced from total hemispherical reflectance and transmittance spectra.

In the near-IR, absorbance lines in silk fibers can be attributed to the combination or overtone modes of various vibrations of the chemical bonds of the constituent peptides [48]. An example of such combination modes are the vibrations of aliphatic C–H observed at 4010 cm${}^{-1}$ due to ν(C–H)${}_{s}$/C-H skeletal vibrations and at 4105 cm${}^{-1}$ due to combined ν(C–H)${}_{as}$/C–H skeletal vibrations. Similarly, the lines at 4050 cm${}^{-1}$ and 4170 cm${}^{-1}$, respectively, are assigned to combination modes of ν(C–H)${}_{s}$/*r*(C–H) and ν(C–H)${}_{as}$/*r*(C–H). The next group of four vibration combinations has similarly been tied to ν(C–H)/δ(C–H) motions in symmetric/symmetric (4250 cm${}^{-1}$), asymmetric/symmetric (4303 cm${}^{-1}$), symmetric/asymmetric (4348 cm${}^{-1}$) and asymmetric/asymmetric (4425 cm${}^{-1}$) configurations, respectively.

The spectral range between 4500–4900 cm${}^{-1}$ is especially informative, since it contains the combinations of various amide lines: Amide A and Amide III (4525 cm${}^{-1}$), Amide B and Amide II (4605 cm${}^{-1}$), Amide A and Amide II (4855 cm${}^{-1}$). Particularly, the Amide A and Amide III combination at 4525 cm${}^{-1}$ has been shown to be associated with β-sheets as it relates to the strength and prevalence of hydrogen bonds [49]. In contrast, Amide A and Amide II at 4855 cm${}^{-1}$ tied to random α-coils [48,49]. Thereby, the larger fraction of β-sheets in *B. mori* over the wild silk variety observed in mid-IR spectra is corroborated by results in the near-IR.

The pronounced peak at 5167 cm${}^{-1}$ is reported as arising due to ν(O–H) and δ(O–H) combined vibrations [48]. Spectral features beyond 5200 cm${}^{-1}$ represent the first and second overtones of various hydrogen bonds. The spectra reveal low scattering losses at visible wavelengths, which would follow $\propto \lambda^{-4}$ Rayleigh scattering scaling. Regenerated silk fibroin can be used to make optical fibers with waveguiding losses <0.1 dB/cm (at 15,800 cm${}^{-1}$, or 633 nm in wavelength) comparable with polymethylmetacrilate (PMMA) and even original silk fibers perform as optical waveguides of 2.9 dB/mm at visible wavelengths [8].

**Figure 9:**
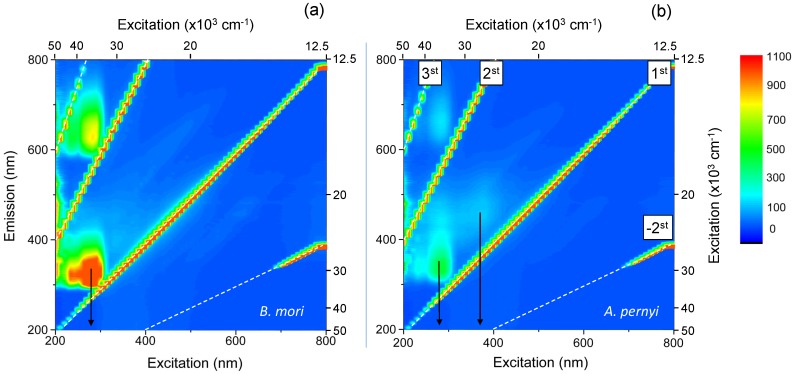
Photoluminescence excitation spectra (PLE) of degummed *B. mori* (**a**) and *A. pernyi* (**b**) silk fibers. The arrow in (**a**) shows center band excitation wavelength ∼35,714 cm${}^{-1}$ (∼280 nm). The first and higher order diffraction artifacts are marked in (**b**). Photoluminescence (PL) at 15,625 cm${}^{-1}$ (640 nm) for the excitation of ∼35,714 cm${}^{-1}$ (∼280 nm) is caused by second order diffraction.

Significant absorption starts to dominate the spectra at UV wavelengths. Figure 9 shows a photoluminescence excitation spectrum (PLE) of degummed *B. mori* and *A. pernyi* fiber specimens. In both cases, a strong PL excitation band centered at the excitation wavelength of 15,800 cm${}^{-1}$ (∼280 nm) was present. It is typical for proteins in natural bio-materials and is related to $\pi ⟶\pi^{*}$ transitions in amino acids that have aromatic rings such as tyrosine, phenylalanine, tryptophan and histidine. Residues of tyrosine in particular have been shown to account for a significant portion of absorption in the 15,800 cm${}^{-1}$ (280 nm) region [50,51]. A remnant of sericin in the degummed fibers has a signature with its absorption band at 42,550–46,500 cm${}^{-1}$ (215–235 nm) (FWHM) wavelengths [52]. The bonds of most other peptides (including the main building blocks of fibroin—alanine, glycine and serine) absorb at wavelengths below 47,620 cm${}^{-1}$ (210 nm); however, due to the abundance and variety of peptide bonds in biomaterials as well as overlapping absorption of other materials, this spectral range presents considerable analytical challenges [53].

In crystalline *A. pernyi* fibers, photoluminescence was relatively stronger than for the *B. mori* fibers and an additional PL band exists for the longer ∼26,315 cm${}^{-1}$ (∼380 nm) excitation wavelengths. It has been reported that similar spectral signatures at ∼28,985 cm${}^{-1}$ (∼345 nm) are observed for sericin solutions extracted from *A. pernyi* silks, with significant variations in UV spectral response of sericin from different silks [51]. The brown pigmentation of certain wild silks has also been hypothesised as resulting from ∼21,740 cm${}^{-1}$ (460 nm) absorbing chromophores created through UV photo-oxidation of tyrosine residues, which may play a part in UV protection of the silk cocoon in its pupal stage [51].

### 3.4. Discussion of the Band Assignment

Absorption band assignments for the wild, domestic and spider silks in IR spectral range have been analysed using a multivariate approach [41] and were used in this study for IR absorption band assignment. It has been demonstrated by numerical modeling and spectral measurements that peptides form secondary structures and have spectrally broad absorption bands in the 100–500 cm${}^{-1}$ spectral region [54]. Typical bond energy in N-methylacetamide (the simplest peptide) is 120 cm${}^{-1}$, assigned to CO···HN intermolecular hydrogen bonding, and 201 cm${}^{-1}$ for the C–N torsional vibration of the peptide bond [54]. However, the assignment to specific bonds is difficult due to substantive broadening occurring as a result of variations in the secondary structure.

Temperature of 77 K corresponds to the thermal energy of 53.5 cm${}^{-1}$. Since the absorption bands did not experience spectral narrowing (Figure 4), it was concluded that those vibrations are intrinsic to the structural components of the crystalline silk fibers and for amorphous fibroin.

It has been demonstrated that Raman active bands observed in scattering of *B. mori* silk at 1085, 1232, and 1667 cm${}^{-1}$ correspond to the absorption of random coils (1085 cm${}^{-1}$) and β-sheets, respectively. Hence, absorption and scattering have similarities in characteristic spectral bands. While bands at 412 and 260 cm${}^{-1}$ are ascribed to the -SO${}_{2}$- moiety [55], the quartet of spectral signatures at around 250, 328, 427, and 553 cm${}^{-1}$, observed in β-sheet rich *B. mori* fibroin films, provide a closer correspondence [39]. The peak at 553 cm${}^{-1}$ is especially instructive as it is observed in (Ala-Gly)${}_{n}$ copolymers, but not in either poly-alanine or poly-glycine. The absence of an equivalent peak in *A. pernyi* fibers, where alanine and glycine rich regions are segregated, further reinforces this assignment. The low energy bands are also found at the silk-I polypeptide recognisably by signatures at about 1415, 1105, 950, 930, 865, 260, and 230 cm${}^{-1}$ [44]. The spectral features of *A. pernyi* fibers at 20 cm${}^{-1}$ and 67 cm${}^{-1}$ (0.6 THz and 2 THz) are, most probably, related to the hydrogen bonding O-H···O where interaction between the neighboring segments define the acoustic response of material in a similar way as in water, where a 70 cm${}^{-1}$ band has been ascribed to the third or fourth neighbor network response [56]. Since *A. pernyi* silk has a smaller amount of β-sheets [31] as compared with the *B. mori* silk which has 60%–65% crystallinity [57], the polymeric network is interacting via hydrogen bond linked segments. This defines spectroscopic differences at a low energy range of T-rays observed in experiments (Figure 1) and explains stretchability and contraction of silk [31]. In addition, significant differences between the spectral signatures of water insoluble fibers and soluble amorphous fibroin, uncovered in the 200–300 cm${}^{-1}$ far-IR spectral region, allow for the inference of important variations in secondary structure of this biopolymer.

## 4. Conclusions

Spectral absorbance of silk over ∼3.8 decades in frequency (3.8/lg2=12.6 octaves) was measured ranging from THz 8 cm${}^{-1}$ (λ= 1.25 mm, f= 0.24 THz) to deep-UV $50\times 10^{3}$ cm${}^{-1}$ (λ= 200 nm, f= 1500 THz). Whereas spectroscopy at the UV and visible ranges provides information about chemical composition, near- and mid-IR offers a wealth of information on the immediate environment of certain bonds, T-ray spectra are exquisitely sensitive to variations in secondary structure, albeit at the cost of specificity. Low energy T-ray bands at ∼243 and ∼229 cm${}^{-1}$, probably related to CO–NH torsional vibrations [39] in domestic and wild degummed silk fibers, respectively, were observed and showed no spectral shift down to 78 K temperature. The difference between domestic and wild silk crystalline fibers was observed at 20 cm${}^{-1}$ and 67 cm${}^{-1}$ (0.6 THz and 2 THz) bands, which correspond to the spectral region of acoustic response in hydrogen bonding networks.

High transmittance of silk up to 35,714 cm${}^{-1}$ (280 nm) wavelengths and its birefringence makes it a promising material for micro-optical applications where polymerisable resists and resins usually have low transmittance. This can be found to be appealing for micro-fluidic and opto-genetic applications using aqueous and bio-tissue ambiance. Water solubility of silk, its biodegradability and in-body desorption are critically important for fabrication of bio-sensor platforms [58], implants, and wearable electronics which are fast developing using silk and can be better understood from spectroscopic analysis over a broad spectral range. Far-IR/THz spectroscopy bolsters the chemical fingerprinting capability afforded by shorter wavelength-based methods, as it is capable of probing the secondary structure of complex protein-based materials even in their natural states. In this study, silkworm spun fibers were investigated as a point for future reference. However, such broad spectrum analysis is useful in investigating the structural hierarchy of silk to guide bottom-up methods for self-assembly of fibroin-based artificial biocompatible functional materials.
